# What are the personal and professional characteristics that distinguish the researchers who publish in high- and low-impact journals? A multi-national web-based survey

**DOI:** 10.3332/ecancer.2017.718

**Published:** 2017-02-07

**Authors:** Carlos Eduardo Paiva, Raphael L C Araujo, Bianca Sakamoto Ribeiro Paiva, Cristiano de Pádua Souza, Flavio Mavignier Cárcano, Marina Moreira Costa, Sérgio Vicente Serrano, João Paulo Nogueira Lima

**Affiliations:** 1 Department of Clinical Oncology, Barretos Cancer Hospital, Barretos, SP, Brazil, 14784-400; 2 Institute of Education and Research/Pio XII Foundation, Barretos Cancer Hospital, Barretos, SP, Brazil, 14784-400; 3 Department of Upper Gastrointestinal and Hepato-Pancreato-Biliary Surgery, Barretos Cancer Hospital, Barretos, SP, Brazil, 14784-400; 4 Barretos School of Health Sciences, Dr Paulo Prata, Barretos, São Paulo, Brazil; 5 Department of Medical Oncology, AC Camargo Cancer Centre, São Paulo-SP, Brazil

**Keywords:** researcher, survey, impact factor, publication process

## Abstract

**Purpose:**

This study identifies the personal and professional profiles of researchers with a greater potential to publish high-impact academic articles.

**Method:**

The study involved conducting an international survey of journal authors using a web-based questionnaire. The survey examined personal characteristics, funding, and the perceived barriers of research quality, work-life balance, and satisfaction and motivation in relation to career. The processes of manuscript writing and journal publication were measured using an online questionnaire that was developed for this study. The responses were compared between the two groups of researchers using logistic regression models.

**Results:**

A total of 269 questionnaires were analysed. The researchers shared some common perceptions; both groups reported that they were seeking recognition (or to be leaders in their areas) rather than financial remuneration. Furthermore, both groups identified time and funding constraints as the main obstacles to their scientific activities. The amount of time that was spent on research activities, having >5 graduate students under supervision, never using text editing services prior to the publication of articles, and living in a developed and English-speaking country were the independent variables that were associated with their article getting a greater chance of publishing in a high-impact journal. In contrast, using one’s own resources to perform studies decreased the chance of publishing in high-impact journals.

**Conclusions:**

The researchers who publish in high-impact journals have distinct profiles compared with the researchers who publish in low-impact journals. English language abilities and the actual amount of time that is dedicated to research and scientific writing, as well as aspects that relate to the availability of financial resources are the factors that are associated with a successful researcher’s profile.

## Introduction

Since evidence-based medicine (EBM) has been in vogue, part of a scientific reputation either for the researcher or institution has been associated with their publication volume and quality. The measurement of the impact and scientific productivity of a researcher has relevant practical implications on achieving titles and better academic positions to increase the chances of obtaining research funding [1, 2]. Several metrics are employed to assess the impact of a journal or of a researcher's production in the scientific community [3, 4]. The number of scientific citations of a given published article is most frequently used as a measured outcome to calculate the overall impact factor (IF) of journals. In addition to assessing the specific impact of an article and scientific journal, IF also makes it possible to measure the impact of a researcher’s work. Although there is controversy regarding some aspects of this measurement [5, 6], the Hirsch index (h-index) has been the most used metric to assess a researcher’s impact [4, 7]. The h-index is defined by how many of a researcher’s publications (Np) have at least h citations each and how many other (*N_p_* – *h*) publications have ≤*h* citations each [4].

Thus, researchers are driven to submit their studies to journals with a high IF and high visibility to seek a greater citation rate for their papers. Tang *et al* demonstrated that the publications of positive outcomes in oncologic clinical trials tend to be accepted in journals with higher IFs which could represent an ‘impact-factor bias’ [8]. This approach generates fierce competition among scientific groups for the few slots that are available in these high-ranking periodicals [9–11]. Considering that the scientific broadcast process can be extremely rigorous and competitive, researchers must allocate their working time to research, clinical, management, and teaching duties, and also researchers may even be compelled to use their leisure time to achieve their research goals. The impact of this requirement on the expectations, personal perceptions, and productivity of researchers is immeasurable. Perhaps this impact is related to the type of work, professional experience, academic level, number of subordinate researchers, and other factors. Therefore, our main objective is to characterise the personal and professional profiles of the researchers who have a greater potential to publish high-impact academic articles.

## Methods

### Study design

A multicentre, cross-sectional study was designed using a web-based questionnaire.

### Selection of journals

The journals in the category of ‘Medicine, General & Internal’, as classified by the Journal Citations Report (JCR) Science Edition 2012 (ISI Web of Knowledge), were selected for this study. In this category, 151 journals were identified with a median IF of 1.067 from which two groups of general medicine journals were selected, namely the journals with a high scientific IF and the journals with a low scientific IF. The high-IF journals were defined as having extremely high (outliers) IFs that ranged between 13.976 and 51.568. The journals with IFs between the 25th and 50th percentiles (IF=0.50–1.067) were considered to be low-IF journals. Initially, the authors noted that some low-IF journals annually published few original articles, but later it was estimated that each journal would sum up to 30–35 articles that could also potentially be evaluated. Thus, considering a total sample of 780 invitations that could be sent to the researchers from the group of low-IF journals, the authors randomly selected 24 journals out of 39.

### Selection of potential participants (researchers)

The corresponding authors of the original articles that were published in the selected journals between January 1, 2013 and July 31, 2013 were included. The corresponding authors from the low-IF journals who had also published articles in high-IF journals in the past five years (as first author, corresponding author, or last author) and/or the authors whose contact emails were unavailable were excluded.

### Development of the questionnaire

We developed the Researcher’s Profile Survey to evaluate the following domains: (1) the personal characteristics of the researchers; (2) the perceptions of the barriers to the development of research; (3) the process of manuscript writing and journal publication; and (4) work-life balance and career satisfaction and motivation.

### Pilot test

The final questionnaire in English (Table S1) was transposed in full to SurveyMonkey (https://pt.surveymonkey.com/) using the editing tools of the programme. The pilot test was performed by emailing the questionnaire in English to eight researchers who speak English fluently (North American, n = 2; Portuguese, n = 1; Brazilian, n = 5) so that they could clarify any possible difficulties that may be encountered in completing the questionnaire.

### Data collection

Each corresponding researcher who was potentially eligible for the study was identified individually after an analysis of the articles by the researcher that were published in the selected journals during the previously established time period. The participants were invited by an email that contained a link to the study page. The first page of the study asked the participants to give their consent prior to them continuing with the questionnaire. The emails were resent to the researchers who did not answer by the seventh and the thirtieth day when necessary. Those researchers who still had not responded by 90 days after the initial invitation were considered to be ‘non-responders’, and it was thus concluded that the non-responding researchers did not want to voluntarily participate in the study. The data collection was conducted between December 2013 and August 2014.

### Analysed variables

#### The personal characteristics of the researchers

This domain investigated the personal characteristics of the researchers who were surveyed, such as age (continuous variable), educational degree (PhD, MSc, research fellow, or post-doctoral fellow), university education (physician or not a physician), time invested in scientific research (<3 years, 3–5 years, 5–10 years, 10–20 years, or >20 years), present work position held, main place of work, time spent on research activities (<10% of time, 10%–25% of time, 25%–50% of time, 50%–75% of time, 75%–90% of time, or >90% of time), and whether the researcher was a member of any collaborative group (yes or no). The variables ‘current official work language’ (English language or not English language) and ‘Gross Domestic Product’ (continuous variable) were created based on the investigated item ‘country of residence’. Regarding the item of ‘how many graduate students are working in collaboration with you?’, the answers ‘6–10’ and ‘more than 10’ were grouped as ‘more than 6’ for statistical purposes. The answer ‘I prefer not to answer’ was considered ‘not available’.

#### Putative barriers to improve research quality

The item ‘what are the regular sources of funding that you rely on for your research activity?’ led to the creation of the following variables: government (yes or no), industry (yes or no), university hospital grant (yes or no), own resources (yes or no), awards and non-governmental funds (yes or no), and other sources of funding (yes or no). The other variable that was evaluated in this domain was ‘What do you believe is the main obstacle to improve the quality of your work?’, which yielded one of the following responses: ‘lack of time’, ‘lack of funding’, ‘lack of support from your institution’, ‘lack of cooperative trained staff’, ‘excess of administrative tasks’, ‘excess of other activities’, or ‘no blocking issues’.

#### Process of manuscript writing and journal publication

In this domain, the analysed variables were ‘use of medical or text editors before submitting a paper to the journal’ (always, very frequently, sometimes, rarely, or never), ‘impact of reputation of the author(s) on acceptance or rejection of a manuscript’ (no impact, minor impact, or relevant impact), and ‘impact of reputation of the institution(s) to which the author(s) are affiliated on acceptance or rejection of a manuscript’ (no impact, minor impact, or relevant impact). The researchers were asked about their knowledge regarding the h-index (yes or no) and their opinion about the ‘adequacy of h-index to measure the excellence of a researcher’ (continuous variable).

#### Work-life balance and career satisfaction and motivation

The analysed variables were how much time after work (during supposedly leisure time) did the researchers address research and spend with family and loved ones. When adjusted to the categorical variables, these answers were divided between <25% and answers >75%. Career satisfaction was also measured (very satisfied, moderately satisfied, or unsatisfied). In the item ‘personal preference for being a leading professional or really well paid’, the answer ‘I prefer not to answer’ was treated as ‘not available’.

### Statistical analyses

The questionnaires with less than 50% of the responses were excluded from the analyses. The frequencies and descriptive statistics are expressed as (%), as medians, as well as 25th and 75th percentiles. The responses were analysed to attempt to characterise a personal-professional profile that was associated with some proportion of the group of researchers. The categorical variables were compared using a chi-squared test or Fisher’s exact test. The continuous variables were compared between the groups of researchers who publish in high- and low-impact journals using the Mann-Whitney test. The variables with p-values <20 were included in the logistic regression analysis (stepwise backward) to identify the independent predictors of publication in high-impact journals. The Hosmer-Lemeshow test was used to evaluate whether the statistical model was appropriate for the analyses. In all of the analyses, a p-value of less than 0.05 was considered to be significant. The statistical programme of SPSS version 20.0 was used (SPSS Inc., Chicago, IL, USA).

### Calculation of the sample size

To estimate the sample size of the researchers who were needed for each of the evaluated groups, the statistical method for dichotomous variables was used [11] ‘In a regular work week, how much time after work–during your supposedly leisure time– do you use to address research (develop studies, review protocols and papers, etc.)?’ was considered the item that best illustrated the study hypothesis; we arbitrarily considered ‘success’ as spending less than 50% of leisure time handling research subjects. Thus, our hypothesis was that there would be a difference of approximately 15% between the groups with the expectation that the success rates of the researchers who publish in high- and low-impact journals would be 75% and 90%, respectively. Considering α = 5% and a test power of (β) = 90%, the minimum estimated sample size was 130 researchers for each group. Thus, for a response rate of 20% to the questionnaires we would need to send the questionnaire to at least 1300 researchers (650 in each group). Considering that some contact details may be outdated, we applied a 20% overhead for the loss of contact emails. Therefore, we planned to contact a final number of 1560 scientists.

## Results

Of the 1561 invitations that were sent using the electronic tool, 280 researchers agreed to participate in the study. Of these researchers, 11 were excluded from the analyses because they responded to less than 50% of the questions on the questionnaire. Thus, the final sample size was 269 participants (overall response rate = 17.2%; low impact n = 121, response rate = 13.4%, and high impact n = 148, response rate = 22.6%). Table S2 details the journals that were evaluated with their respective response rates.

The median age of the participants was 45 years old (range: 26–90 years old). Regarding their educational background, the majority were medical doctors (n = 184, 68.4%), but members of other professional areas also participated in the study (biology n = 15; pharmacy n = 5; biomedicine n = 20; psychology n = 11; statistics n = 11; nursing, n = 6; dentistry n = 3; epidemiology n = 9; public health n = 7; chemistry n = 6). Eight participants had two distinct educational backgrounds.

### Characteristics of the investigated researchers

The two evaluated groups (researchers who published in high- or low-impact journals) actually represented researchers with distinct publication records who had statistically significant differences in the medians of their h-indices (15.3 and 3, respectively, p<0.001). The number of publications that were indexed in the ISI Web of Knowledge was also different (high-impact researchers, median = 57 and low-impact researchers, median=10, p<0.001; Table 1).

The researchers who published in high-impact journals were less frequently medical doctors (p = 0.030) and more often had PhDs (p = 0.031) and were involved in mentoring graduate students (p = 0.008). High-impact researchers more commonly worked at academic institutions (94.6% compared with 81.8%), most notably at universities (48.3% compared with 28.6%), and less frequently in non-academic services (0% compared with 10.1%). The researchers in the high-impact group tended to more frequently be leading researchers/department leaders (21.1% compared with 14.2%) and head professors (32.7% compared with 13.3%).

Researchers who publish in high-impact journals were older than their low-impact counterparts (p = 0.004). However, not only age but also time in academia was different because researchers who published in high-impact journals have longer time periods devoted to research (p<0.001). Moreover, researchers who published in high-impact journals more frequently lived in countries that were considered wealthier (p = <0.001) and whose official language was English (p<0.001) (Table 1).

### Funding and perceived barriers of research quality

The participants in both groups had rather similar perceptions of the barriers that hindered research. Nearly 90% researchers who publish in high-impact journals and 95% of researchers who publish in low-impact journals face some type of obstacle to conduct their work. The majority of the responders from both groups complained about difficulties because of lack of time (80 of 263, 30.4% in total) and insufficient financial support (73 of 263, 27.8% in total).

We posited that funding would be a major issue in conducting proper research, and we inquired about the main features of funding. Absolute figures were not asked. Interestingly, the source of funding was different between the groups. The researchers who published in high-impact journals received more frequent funding from the government (79.1% compared with 44.5%, p<0.001), industry (24.3% compared with 11.6%, p = 0.007) and non-governmental awards and funds (50% compared with 20.7%, p<0.001), whereas one’s own financial resources were the key source for the authors who published in low-impact journals (5.4% compared with 33.9%, p<0.001). A detailed comparison of the groups is demonstrated in Table 2.

### Work-life balance and career satisfaction and motivation

We asked the researchers to quantify the time that they spent with family, that separately, and the estimated leisure time that they spent on research affairs. The distribution of the time that was spent with family was similar between the groups, and approximately 40% of both groups affirmed that they spent more than half of their free time with their loved ones. However, the researchers who published in high-impact journals dedicated a larger portion of their leisure time to research activities than the researchers who published in low-impact journals (p = 0.003).

When questioned regarding their preference between being very well financially compensated or being considered leaders in their areas, the majority of the participants declared that they would prefer to be a leader (204 of 240, 85%), with no apparent difference between the groups. Notwithstanding, the researchers who published in high-impact journals significantly considered themselves more satisfied with their present work situation than the researchers who published in low-impact journals (p<0.001) (Table 3).

### Processes of manuscript writing, journal publication, and research quality assessment

The majority of the researchers who published in high-impact journals stated that they rarely or never used text editing services prior to submitting their manuscripts for publication; in contrast, the researchers who published in low-impact journals used these services with greater frequency (p<0.001) (Table 4).

When asked whether the reputation of the author or the institution impacts the likelihood of acceptance of a manuscript, nearly 60% of the interviewees of both groups believed that these factors may be relevant.

In general, researchers classified the capacity of the h-index to adequately identify the excellence of research as only average (median score of 5 on a scale from 0 –10). Again, there were no differences in the perception between the groups regarding this aspect. However, more researchers who published in high-impact journals were aware of the h-index than their low-impact counterparts (69.7% compared with 54.8%, p = 0.014) (Table 4).

### Profile of success as an author of high-impact papers

We explored which features are most likely connected to the high-impact researcher profile by performing a logistic regression analysis. The variable ‘Time spent on research activities’ was an independent predictive variable, with the chance of ever publishing in a high-impact journal increasing gradually as the researcher dedicated more time to research. This variable reached odds ratios (ORs) of 23.2 and 36.0 when the researchers used 75–90% and >90%, respectively of their work time for research activities (Table 5).

Having more than five graduate students under supervision (OR = 4.1), never using text editing services prior to the publication of articles (OR = 10.90), and living in a country whose official language is English (OR = 2.85) that has a higher per capita gross domestic product (OR = 1.05) were the independent variables that were associated with a greater chance of being published in a high-impact journal. However, living in a country that has a higher per capita gross domestic product had a borderline relevance. In contrast, using one’s own resources to perform studies was an independent variable that decreased the chance of publishing in a high-impact journal (OR = 0.22) (Table 5).

## Discussion

Researchers in the healthcare arena may receive academic recognition for various skills, such as their leadership and professional activities, honours and awards, research grants, teaching and mentoring/advising activities, recognised scientific integrity, participation in breakthrough discoveries, and even the ability of forming research networks [12]. However, the criterion that most objectively evaluates the potential of a researcher is his record of scientific publications in peer-reviewed journals. We investigated the personal and professional characteristics of the researchers who publish in medical journals; the authors who had published in high-impact journals were compared with the authors who had published in journals with a lower scientific impact. The calculation of the h-index and the tallying of the number of publications in the journals that were indexed by the ISI Web of Knowledge for each researcher confirmed that these groups were in fact distinct. Nevertheless, other differences emerged.

### Principal findings

The authors who published in high-impact journals who answered our survey were senior researchers, in a higher age range, held leadership positions in their departments, worked in universities, had more previous research experience, and had more graduate students under their supervision. Also a higher percentage of authors in this category held PhD degrees. This assemblage is precisely the profile that is traditionally associated with greater academic prestige among researchers. Interestingly among these factors, an intense mentoring activity, here depicted as tutoring more than five graduate students, was the most significant factor on multivariate analysis. Our data suggest that a direct commitment with research, exemplified by tutoring junior researchers, is more relevant than occupying a high rank position in the hierarchy of an academic institution.

Time available to work specifically on research was the most important variable that was associated with a greater chance of publication in a high-impact journal. The chances were almost 36 times higher if a researcher dedicated more than 90% of his working time to research when compared with 10%. Additionally, many of the respondents used their leisure time for scientific duties. Protected research time was one of the factors that was associated with the career success of researchers in a previous study [13]. These findings underscore how time-consuming research is for the researchers of medical science.

The researchers who published in low-impact journals may face particular challenges in publishing their work, including funding obstacles and difficulty in writing manuscripts. It is conceivable that an author would only use self-funding if he failed to obtain a grant from a formal source. Therefore, the finding that self-funding is linked to lower chances of publishing in high visibility journals may suggest that these studies were not deemed worthy of obtaining a grant from formal sources or that the grant offer was severely limited. This question of competition for funding is extremely important at present because intense competition may be undermining scientific integrity [14].

Living in a country where English is the official language was associated with almost a three-fold greater chance of publishing in a high-impact journal, and this factor was more relevant than being located in a wealthy country. This result corroborates the findings of Tang *et al* which showed that the publication of clinical trials in oncology can address some biased trends such as the presence of positive outcomes, adjuvant treatment, uncommon cancers, and nationality (English speakers compared with non-English speakers) [8]. Thus, the risk of ‘editorial bias’ cannot be excluded in oncology journals. We speculate that a command of the English language, more supportive institutions, and the availability of funding make researchers’ work more competitive. This speculation would also explain why medical and copyediting use is increasing among less-experienced researchers [15, 16]. Encouraging findings from a national career development programne have been published by Bruce *et al* [17]. The development of grant preparation and time management skills and the opportunity of consulting with statistical experts made young researchers more competitive for obtaining research funding.

Although many differences between high- and low-impact authors were detected, several similarities between the two groups should be emphasised. Apparently, researchers in the medical field seek recognition (or to be leaders in their areas) rather than financial remuneration. A previous study evaluated successful orthopaedic surgeons, and it showed that earning money was not the most important source of motivation [18]. Furthermore, both groups identified time and funding constraints as the main obstacles to their scientific activities which confirmed previous results [19, 20]. Both groups also believed that the name or fame of the institution or the researcher would have a relevant role in the publishing outcome of the submitted paper. Therefore, these results seem to reveal a common profile of a researcher in terms of their perceptions and ambitions in the medical arena.

### Strengths and weaknesses of the study

One major limitation of this study was the low response rate. Therefore, we cannot exclude non-responder bias. Considering that researchers are generally very busy–a fact that is confirmed by the perception of a lack of time as a barrier in this study–we had already predicted a low response rate (~20%). Interestingly, the researchers who published in high-impact journals showed a higher response rate than the researchers who published in low-impact journals (22.6% compared with 13.4%). This suggests greater interest and also curiosity concerning this study is from the researchers who published in high-impact journals.

Based on our findings and considering that this is a cross-sectional study, it is not possible to generate causal relationships. For instance, we found that the researchers who supervise more than five graduate students were likely to have published in high-impact journals; however, perhaps because of their high publication skills, these researchers can attract more students to supervise. Similarly, we determined that the researchers who dedicate more than 90% of their working time to research were more likely to publish their papers in high-impact journals than the researchers who dedicate less than 10% of their working time to research. This finding can suggest a bias because the researchers who spend 90% of their time may be career researchers, while the researchers who spend less than 10% of their time are probably clinicians who also do research. Further studies are needed to confirm our findings.

Another limitation was that we did not evaluate personal qualities such as critical attitude, independence, creativity, curiosity, and determination, which are already known to relate to a greater potential to perform research [21]. However, the inclusion of more items in our questionnaire could have further decreased the response rate. Despite these limitations, we can emphasise the originality of our findings that show the profiles of researchers according to the impact of their publications and facility, as well as their satisfaction with their careers. Moreover, our methodological strategy identified two groups of researchers with distinct publication records.

### Implications for researchers and institutions

Institutions and researchers who aim to publish their research in high-impact publications attempt to improve scientific and clinical practice, and the key points to attain this goal were addressed in our survey. High-quality research is a time- and fund-demanding activity, and all steps in the process should be properly addressed. On practical grounds, enhancing writing and article editing knowledge also seems to have paramount importance. Similarly, improvement should be sought concerning how to apply for grants. Considering that there is a ubiquitous shortage of time, offering more time or at least a more rational use of time to researchers may be an effective approach to improve the results.

## Conclusion

The researchers in the medical arena who publish in high-impact journals have distinct profiles compared with the researchers who publish in low-impact journals. The amount of time that is dedicated to research, scientific writing, and English language abilities, as well as the aspects that relate to the availability of financial resources are factors that are associated with a successful researcher’s profile. However, the researchers who published in high- and low-impact journals share some opinions on career targets.

## Conflicts of interest

No funding. The authors have no conflicts of interest to declare.

## Disclosure of results at a meeting

None

## Ethical approval

This study complied with the ethical standards of the Declaration of Helsinki and Brazilian National Health Council resolution no. 466/2012. It was approved by the Research Ethics Committee of the Cancer Hospital of Barretos (Opinion no. 468.654). After having access to an online informed consent form, all of the participants voluntarily agreed to participate in this study.

## Supplemental material

## Figures and Tables

**Table 1.** Characteristics of the investigated researchers.

Variable	High impact n (%)	Low impact n (%)	p-value
*Personal h-index (median; p25–p75)*	15.5 (7.0–26.7)	3 (1.0–7.0)	<0.001
*ISI indexed publications1 (median; p25–p75)*	57 (24.0–118.2)	10 (4.0–30.5)	<0.001
*Age (years)*			0.004
Median	47	43	
P25–p75	39.2–57.7	35.0–52.0	
*Physician*			0.030
Yes	93 (62.8)	91 (75.2)	
No	55 (37.2)	30 (24.8)	
*MSc*			0.401
Yes	37 (25.0)	25 (20.7)	
No	111 (75.0)	96 (79.3)	
*PhD*			0.031
Yes	88 (59.5)	56 (46.3)	
No	60 (40.5)	65 (53.7)	
*Post doctoral fellow*			0.880
Yes	13 (8.8)	10 (8.3)	
No	135 (91.2)	111 (91.7)	
*Research fellow*			0.495
Yes	8 (5.4)	9 (7.4)	
No	140 (94.6)	112 (92.6)	
*Current official work language*			<0.001
English language	116 (78.4)	42 (34.7)	
Other language	32 (21.6)	79 (65.3)	
Time involved in scientific research			<0.001^*^
<3 years	1 (0.7)	8 (6.6)	
3–5 years	14 (9.5)	26 (21.5)	
5–10 years	24 (16.3)	38 (31.4)	
10–20 years	45 (30.6)	21 (17.4)	
>20 years	63 (42.9)	28 (23.1)	
NA	1	0	
*Position hold^2^*			<0.001
Chief researcher, head of department	31 (21.1)	17 (14.2)	
Assistant researcher	7 (4.8)	12 (10.0)	
Chief Professor (reader, senior lecturer)	48 (32.7)	16 (13.3)	
Assistant professor	22 (15.0)	24 (20.0)	
Non-academic professional	8 (5.4)	23 (19.2)	
Other	31 (21.1)	28 (23.3)	
NA	1	1	
*Member of a research Collaborative Group*			0.114
Yes	96 (65.3)	67 (55.8)	
No	51 (34.7)	53 (44.2)	
NA	1	1	
*Time spent on research activities*			<0.001
<10% of time	3 (2.1)	21 (17.5)	
10–25% of time	13 (8.9)	41 (34.2)	
25–50% of time	28 (19.2)	25 (20.8)	
50–75% of time	47 (32.2)	19 (15.8)	
75–90% of time	38 (26.0)	10 (8.3)	
>90% of time	17 (11.6)	4 (3.3)	
NA	2	1	
*Main place of work*			<0.001^*^
Teaching hospital	55 (37.4)	56 (47.1)	
Government	6 (4.1)	1 (0.8)	
University	71 (48.3)	34 (28.6)	
Private practice	0 (0)	12 (10.1)	
Some research centre	4 (2.7)	3 (2.5)	
Other	11 (7.5)	13 (10.9)	
NA	1	2	
*Number of post-graduate students working with*			0.008
None	28 (19.2)	42 (35.9)	
1–5	93 (63.7)	62 (53.0)	
>5	25 (17.1)	13 (11.1)	
NA	2	4	
*GDP × 10*^3^* (median, p25–p75)^3^*	40.9 (38.0–53.1)	35.0 (13.0–39.1)	<0.001

*Abbreviations*: IQR = interquartile range; GDP = Gross Domestic Product; NA=not available.

¹Indexed in Web of Knowledge (ISI).

² consider the one that occupies most of your time.

³ Values calculated per capita in dollars.

* Fisher exact test.

**Table 2.** Perceived barriers to improve research quality.

Variable	High impact n (%)	Low impact n (%)	p-value
*Main obstacle to improve the quality of research?*			0.511
Lack of time	49 (33.6)	31 (26.5)	
Lack of funding	35 (24.0)	38 (32.5)	
Lack of support from your institution	7 (4.8)	8 (6.8)	
Lack of cooperative trained staff	13 (8.9)	8 (6.8)	
Excess of administrative tasks	12 (8.2)	8 (6.8)	
Excess of other activities	15 (10.3)	16 (13.7)	
No blocking issues	15 (10.3)	8 (6.8)	
NA	2	4	
*Regular sources of funding for the research activity*			
Government			<0.001
Yes	117 (79.1)	54 (44.6)	
No	31 (20.9)	67 (55.4)	
Industry			0.007
Yes	36 (24.3)	14 (11.6)	
No	112 (75.7)	107 (88.4)	
University Hospital Grant			0.453
Yes	31 (29.9)	30 (24.8)	
No	117 (79.1)	91 (75.2)	
Own resources			<0.001
Yes	8 (5.4)	41 (33.9)	
No	140 (94.6)	80 (66.1)	
Awards and non-governmental funds			<0.001
Yes	74 (50.0)	25 (20.7)	
No	74 (50.0)	96 (79.3)	
Other sources of funding			0.304
Yes	15 (10.1)	8 (6.6)	
No	133 (89.9)	113 (93.4)	

*Abbreviations*: NA = not available.

**Table 3.** Balance work-life, satisfaction, and motivation in relation to career.

Question	High impact n (%)	Low impact n (%)	p-value
*Time after work – during supposedly leisure time – dealing with research*			0.003
<25% of the free time	65 (44.8)	72 (62.6)	
25–50% of the free time	49 (33.8)	35 (30.4)	
50–75% of the free time	16 (11.0)	6 (5.2)	
>75 of the free time	15 (10.3)	2 (1.7)	
NA	3	6	
*Time after work–during supposedly leisure time–spending with the family and beloved ones*			0.763
<25% of the free time	45 (31.0)	42 (37.2)	
25–50% of the free time	40 (27.6)	30 (26.5)	
50–75% of the free time	34 (23.4)	23 (20.4)	
>75 of the free time	26 (17.9)	18 (15.9)	
NA	3	8	
*Personal preference for being a leading professional or really well paid*			0.381
Leading professional	118 (86.2)	86 (82.7)	
Really well paid	18 (13.2)	18 (17.3)	
NA	12	17	
*Satisfaction with career*			<0.001
Very satisfied	68 (47.9)	30 (26.8)	
Moderately satisfied	71 (50.0)	70 (62.5)	
Unsatisfied	3 (2.1)	12 (10.7)	
NA	6	9	

*Abbreviations*: NA = not available.

**Table 4.** Process of manuscript writing and journal publication according to the different published groups.

Question	High impact n (%)	Low impact n (%)	p-value
*Use of medical or text editors before submitting a paper to a journal*			<0.001
Always	2 (1.4)	14 (12.7)	
Very frequently	4 (2.8)	18 (16.4)	
Sometimes	9 (6.3)	20 (18.2)	
Rarely	22 (15.3)	20 (18.2)	
Never	107 (74.3)	38 (34.5)	
NA	4	11	
*Impact of reputation of the author(s) on acceptance or rejection of a manuscript*			0.363^*^
There is no impact on the final outcome	4 (2.9)	7 (6.7)	
It may have a minor impact	46 (32.9)	32 (30.5)	
It can make a relevant difference	90 (64.3)	66 (62.9)	
NA	8	16	
*Impact of reputation of the institution(s) to which the author(s) are affiliated on acceptance or rejection of a manuscript*			0.072
There is no impact on the final outcome	10 (7.1)	11 (10.7)	
It may have a minor impact	65 (46.4)	33 (32.0)	
It can make a relevant difference	65 (46.4)	59 (57.3)	
NA	8	18	
*Know the metric h-index*			0.014
Yes	101 (69.7)	63 (54.8)	
No	44 (30.3)	52 (45.2)	
NA	3	6	
*Adequacy of H-index to measure the excellence of a researcher^1^ (median; p25–p75)*	5 (3.75–7)	5 (3–7)	0.513

*Abbreviations:* h-index = Hirsch index; NA = not available.

1 Mark from 0 to 10, being 0 not at all and 10 yes, absolutely.

* Fisher exact test.

**Table 5.** Independent predictors of being a high-impact researcher.

Variable	OR	95% CI	p-value
Time spent on research activities			
<10% of time	1 (reference)		
10–25% of time	3.88	0.72–20.80	0.113
25–50% of time	11.76	2.16–64.01	0.004
50–75% of time	11.24	2.21–57.02	0.004
75–90% of time	23.19	3.84–140.09	0.001
>90% of time	35.99	3.89–332.62	0.002
*Regular sources of funding for the research activity: own resources*			
No	1 (reference)		
Yes	0.22	0.08–0.66	0.006
*Number of post-graduate students working with*			0.072
None	1 (reference)		
1–5	1.63	0.64–4.12	0.303
>5	4.10	1.09–15.38	0.037
*Use of medical or text editors before submitting a paper to a journal*			
Always	1 (reference)		
Very frequently	1.46	0.18–12.18	0.725
Sometimes	3.40	0.44–26.36	0.242
Rarely	5.81	0.80–42.21	0.082
Never	10.90	1.73–68.74	0.011
*GDP^1^ × 10^3^(per 1000 dollars)*	1.05	1.02–1.08	0.001
*Current official language*			
Other language	1 (reference)		
English language	2.85	1.24–6.54	0.014

*Abbreviations*: GDP = Gross Domestic Product.

**Table S1.** Researchers’ profile survey.

1. What is your age? (Please enter your age in numbers):________________________
2. Country of birth? ____________________
3. Country of residence? ___________________
4. What is your educational degree? (you can choose more than one) University graduation MSc PhD Research Fellow Post-doctoral fellow Other (please specify)
5. What is your University education? Medicine (MD, BCh, MB) Biology Pharmacology Biomedical sciences Psychology Statistics Nursing Other (please specify) ____________________
6. How long have you been involved with scientific research? <3 years 3–5 years 5–10 years 10–20 years >20 years I prefer not to answer
7. What is the present position you hold? (consider the one that occupies most of your time) Chief researcher, head of department Assistant researcher Chief Professor (reader, senior lecturer) Assistant professor Non-academic professional Other (please specify)
8. Are you a member of any research Collaborative Group? Yes No
9. In a regular week, between research, working and/or teaching activities, how much of your time do you spend on research activities? <10% of time 10–25% of time 25–50% of time 50–75% of time 75% – 90% of time >90% of time I prefer not to answer
10. What is your main place of work? Teaching hospital Pharmaceutical company University Private practice Other (please specify)
11. What are the regular sources of funding that you rely on for your research activity? (you can choose more than one)? Government Industry University Hospital grant Own resources (from your pocket) Awards and non-governmental agency funds Other (please specify)
12. What do you believe is the main obstacle to improve the quality of your work? Lack of time Lack of funding Lack of support from your institution Lack of cooperative trained staff Excess of administrative tasks Excess of other activities I do not have issues blocking my scientific activity
13. How many post-graduate students are working in collaboration with you? None 1–5 6–10 More than 10
14. In a regular week of work, how much time after work–during your supposedly leisure time–do you use to deal with research (develop studies, review protocols and papers etc)? <10% of the free time 10–25% of the free time 25–50% of the free time 50–75% of the free time 75%–90% of the free time >90% of the free time I prefer not to answer
15. In a regular week of work, how much time after work–during your supposedly leisure time–do you spend with your family and beloved ones? <10% of time 10–25% of time 25–50% of time 50–75% of time 75–90%of time >90% of time I prefer not to answer
16. How frequently do you rely on the services of medical or text editors before submitting a paper to a journal? Always Very frequently Sometimes Rarely Never I prefer not to answer
17. Do you know what is the research metric named h-index (Hirsch index)? Yes No
18. In your opinion, is the H-index an adequate metric to measure the excellence of a researcher? Not at all 0,1,2,3,4,5,6,7,8,9,10 Yes, absolutely
19. Do you believe that the reputation of the author(s) has any role in the final decision of acceptance or rejection of a manuscript? I believe there is no impact on the final outcome I believe that it may have a minor impact I believe that it can make a relevant difference, increasing the chances of having the paper accepted. I do not know
20. Do you believe that the reputation of the institution(s) to which the author(s) are affiliated has any role in the final decision of acceptance or rejection of a manuscript? I believe there is no impact on the final outcome I believe that it may have a minor impact I believe that it can make a relevant difference, increasing the chances of having the paper accepted. I do not know
21. If you were asked to choose between ‘being recognised as a leading professional’ or ‘being really well paid’, what would be your choice? Being a leading professional Being really well paid I prefer not to answer
22. Are you happy with the course of your career? Very much, I would not imagine myself in other activity Yes, but it could be better No, I would expect something else I prefer not to answer
23. Think about your life as a whole. What grade would you give to your sense of GLOBAL WELL-BEING during the last month? *(not included in the analyses)* Not at all 0,1,2,3,4,5,6,7,8,9,10 Yes, absolutely
24. Think about your life as a whole. What grade would you give to your sense of EMOTIONAL WELL-BEING during the last month? *(not included in the analyses)* Not at all 0,1,2,3,4,5,6,7,8,9,10 Yes, absolutely
25. Think about your life as a whole. What grade would you give to your sense of PHYSICAL WELL-BEING during the last month? *(not included in the analyses)* Not at all 0,1,2,3,4,5,6,7,8,9,10 Yes, absolutely
26. Think about your life as a whole. What grade would you give to your sense of SOCIAL WELL-BEING during the last month? *(not included in the analyses)* Not at all 0,1,2,3,4,5,6,7,8,9,10 Yes, absolutely

**Table S2.** Number of invitations, evaluable responses, and response rates per journal.

Impact	Journal title	Impact factor^¹^	Invitation	Evaluable responses	RR (%)
*Low*					
	Arch Med Sci	1.067	42	3	7.1
	Sao Paulo Med J	0.588	29	8	27.6
	Saudi Med J	0.619	40	5	12.5
	Aten Primaria	0.957	36	6	16.7
	Rev Med Interne	0.899	43	4	9.3
	Acta Clin Belg	0.589	34	3	8.8
	Hippokratia	0.589	36	6	16.7
	Intern Med	0.973	42	3	7.14
	Med Princ Pract	0.963	20	3	15.0
	Wien Klin Wochenschr	0.813	25	4	16.0
	South Med J	0.915	32	4	12.5
	Eur J Gen Pract	0.741	21	3	14.3
	Minerva Med	0.771	23	2	8.7
	J Natl Med Assoc	0.914	30	3	10.0
	J Formos Med Assoc	1.000	23	3	13.0
	Isr Med Assoc J	0.978	32	4	12.5
	Irish J Med Sci	0.506	31	6	19.4
	J Fam Pract	0.669	28	4	14.3
	Chin Med J (Engl)	0.901	54	4	7.4
	J Res Med Sci	0.684	67	11	16.4
	Aust Fam Physician	0.705	45	5	11.1
	Dan Med J	0.923	73	12	16.4
	Presse Med	0.867	77	12	15.6
	Natl Med J India	0.908	23	3	13.0
	Total		906	121	13.4
*High*					
	Ann Intern Med	13.976	76	18	23.7
	Lancet	39.060	127	23	18.1
	PLoS Med	15.253	85	16	18.8
	JAMA	29.978	123	24	19.5
	N Engl J Med	51.658	135	29	21.5
	BMJ	17.215	109	38	34.9
	Total		655	148	22.6

*Abbreviation*: RR = response rate.

1 Journal Citations Report Science Edition (JCR 2012).
